# Cognitive remediation therapy (CRT) as a treatment enhancer of eating disorders and obsessive compulsive disorders: study protocol for a randomized controlled trial

**DOI:** 10.1186/s12888-016-1109-x

**Published:** 2016-11-10

**Authors:** Boris van Passel, Unna Danner, Alexandra Dingemans, Eric van Furth, Lot Sternheim, Annemarie van Elburg, Agnes van Minnen, Marcel van den Hout, Gert-Jan Hendriks, Daniëlle Cath

**Affiliations:** 1 Pro Persona, Centre for Anxiety Disorders Overwaal, Institution for Integrated Mental Health Care, Pastoor van Laakstraat 48, Lent, Nijmegen, 6663 CB The Netherlands; 2 Altrecht Eating Disorders Rintveld, Oude Arnhemseweg 260, Zeist, 3705 BK The Netherlands; 3 Rivierduinen Eating Disorders Ursula, Sandifortdreef 19, Leiden, 2333 ZZ The Netherlands; 4 Department of Psychiatry, Leiden University Medical Centre, Einthovenweg 20, Leiden, 2333 ZC The Netherlands; 5 Altrecht Academic Anxiety Centre, Nieuwe Houtenseweg 12, Utrecht, 3524 SH The Netherlands; 6 Department of Clinical and Health Psychology, Utrecht University, Heidelberglaan 1, Utrecht, 3584 CS The Netherlands; 7 Behavioural Science Institute, Radboud University, Montessorilaan 3, Nijmegen, 6525 HR The Netherlands

**Keywords:** Cognitive remediation, Anorexia nervosa, Obsessive compulsive disorder, Psychological treatment

## Abstract

**Background:**

Anorexia nervosa (AN) and Obsessive Compulsive Disorder (OCD) are among the most incapacitating and costly of mental disorders. Cognitive Behaviour Therapy (CBT), medication, and combination regimens, to which in AN personalised guidance on weight control is added, are moderately successful, leaving room for more effective treatment algorithms. An underlying deficit which the two disorders share is cognitive inflexibility, a trait that is likely to impede treatment engagement and reduce patients’ ability to benefit from treatment. Cognitive remediation therapy (CRT) is an easy-to-use intervention aimed at reducing cognitive inflexibility and thereby enhancing treatment outcome, which we aim to test in a controled study.

**Methods:**

In a randomized-controlled multicenter clinical trial 64 adult patients with AN and 64 with OCD are randomized to 10 bi-weekly sessions with either CRT or a control condition, after which Treatment As Usual (TAU) is started. All patients are evaluated during single-blind assessments at baseline, post-CRT/control intervention, and after 6 months. Indices of treatment effect are disorder-specific symptom severity, quality of life, and cost-effectivity. Also, moderators and mediators of treatment effects will be studied.

**Discussion:**

To our knowledge, this is the first randomized controlled trial using an control condition evaluating the efficacy and effectiveness of CRT as a treatment enhancer preceding TAU for AN, and the first study to investigate CRT in OCD, moreover taking cost-effectiveness of CRT in AN and OCD into account.

**Trial registration:**

The Netherlands Trial Register NTR3865. Registered 20 february 2013.

## Background

Anorexia nervosa (AN) is a severely debilitating eating disorder characterized by self-starvation with both negative physiological and psychological effects. Individuals with AN assign extreme over-importance to body weight and shape, making it central to their self-evaluation, and develop an abnormal body-image perception.

Obsessive Compulsive Disorder (OCD) is characterized by recurrent obsessions and compulsions that cause marked distress and interfere with daily functioning [1]. More specifically, obsessions are defined as intrusive, repetitive thoughts, images, or impulses, and compulsions as purposeful, repetitive overt and covert behaviours performed to relieve obsessional distress.

Several studies have investigated potential relationships between AN and OCD based on the phenotypic features the two disorders have in common, such as repetitive and ritualistic behaviours, excessive habit formation, and cognitive rigidity [2]. Interestingly, several studies demonstrated rates of AN among OCD patients, and reversely rates of OC symptoms among AN patients to be higher than expected by chance, with OCD frequencies ranging between 9,5 and 62 % in patients with AN, and anorexia nervosa-rates up to 11–13 % in clinical OCD populations [3–8].

Further, there is accumulating evidence that patients suffering from AN and OCD share specific inefficiencies in executive functioning affecting attention processes, set-shifting/cognitive flexibility, processing speed, visuospatial abilities, inhibition of ongoing cognitive and motor responses, and working memory [9–14]. Specific inefficiencies in executive functioning are central to the development and maintenance of obsessions and compulsions as seen in both AN and OCD [15, 16].

It is suggested that particularly cognitive inflexibility maintains and exacerbates compulsive behaviours in both AN and OCD [17, 18]. Some degree of cognitive inflexibility is also found in first-degree relatives [19–21], i.e. a difficulty to shift to a different thought or action according to changes in a situation [22]. These inefficiencies are associated with the neurobiological (fronto-striatal) abnormalities associated with repetitive behaviours in AN and OCD [23]. Thus, patients seem to rely heavily on low-energy but highly automated and stereotyped repetitive behaviours, at the cost of higher-energy but more diverse goal-directed and flexible behaviours [24].

Furthermore, individuals with AN show a cognitive style in which there is a bias towards local or detailed-focus processing of information over the natural tendency to integrate information into a for the person relevant context [25]. In line with this, individuals with OCD show inefficiencies in global information processing and a detailed and less systematic organizational strategy [26, 27].

Finally and consistent with previous research, adults suffering from AN and OCD show specific visuospatial memory inefficiencies [26, 28, 29].

Arguably, these inefficiencies may prevent patients from successfully engaging in therapy and may decrease both treatment motivation and the efficacy of psychological interventions.

### Symptom-based treatment for OCD and AN

Treatment schemes for AN and OCD are generally aimed at symptom reduction. Standard treatment of adult AN consists of a combination of renourishment and psychotherapy. Clinical effects of either supportive clinical management, CBT, focal psychodynamic therapy, family therapy, or interpersonal psychotherapy are comparable [30–32]. Only 46% of patients recover fully, one third achieves partial improvement (showing residual symptoms), and 20% remains chronically ill [33]. One significant challenge in the treatment of individuals with AN concerns the ambivalence towards treatment. Intrinsic motivation to enable behavioural changes is often low [34].

According to European guidelines, the treatment of OCD should typically encompass CBT usually in combination with pharmacotherapy, specifically selective serotonin reuptake inhibitors (SSRIs) [35, 36]. Despite reasonable effect sizes (Cohen’s d around 1.0) [37–39], only 22% of OCD patients having received CBT recovers completely [37]. In a recent meta-analysis, Sharma et al. [40] found that 47% of all patients with OCD (treated or untreated) do not achieve complete remission. Thus, rates of incomplete recovery and treatment resistance remain relatively high [41, 42].

Moreover, in OCD non-compliance as to pharmacotherapy is common, as is dropout from CBT (with attrition rates between 13 and 27%) [43–47]. Dropout rates from treatment in outpatients with AN are even higher (ranging from 48 to 100%) [48].

Clearly, optimizing therapy results for AN and OCD remains a major challenge for both researchers and therapists. In the past decades various attempts have been made to develop supportive interventions to enhance adherence and effectivety, with an increasing interest in approaches that target underlying inefficiencies instead of the main symptoms of the disorder itself.

The relatively low success rates of guideline-directed therapies for AN and OCD, together with the inefficiencies in executive functioning that sufferers seem to share, have prompted the notion that patients might benefit from a training module that addresses and remediates these underlying inefficiencies.

### Cognitive remediation therapy

Recently, a cognitive intervention, i.e. Cognitive Remediation Therapy (CRT) has been adapted for eating disorders including AN, which specifically focuses on improving the cognitive inflexibility and organizational inefficiencies described above [49, 50]. CRT was originally developed for the treatment of patients with traumatic brain injuries but has also been applied in patients with attention deficit hyperactivity disorder (ADHD), learning disabilities [51] and schizophrenia. Although in schizophrenia results have been mixed, symptom reduction as well as improvement of cognitive and social functioning, and normalisation of neural activity have been described [52, 53]. CRT aims at improving cognitive flexibility, central coherence, global information processing, reducing perfectionism and improving the awareness of dysfunctional thinking styles.

In CRT for AN, patients carry out cognitive excercies and are encouraged to reflect on their typical, maladaptive daily-life strategies, and to consider and practice helpful alternatives, for instance by adopting more global ‘bigger-picture’ thinking strategies and more goal-directed behaviours. A recent meta-analysis on all available case reports has shown that CRT improves the cognitive performance of patients with severe AN and that the intervention is feasible and well tolerated [54]. Three recent randomized controlled trials (RCTs) similarly found evidence of its effectiveness in AN [55–57]. Dingemans et al. [57] demonstrated CRT preceding TAU to be more effective than TAU alone: the intervention boosted improvement in terms of ED-specific health-related quality of life (QoL) indices (Cohen’s d: 1.36), while ED-related psychopathology was lower at follow-up than it was following TAU alone (Cohen’s d: 0.99). Patients with relatively poor baseline set-shifting abilities showed greater long-term improvements in quality of life in the CRT group when compared to the control group. The authors accordingly concluded that CRT shows promise as a treatment enhancer. Although CRT has not directly been studied in OCD, two RCTs in OCD have tested similar approaches in OCD. Starting from the hypothesis of strengthening executive functions to foster later symptom reduction, Buhlman et al. [58] investigated a cognitive retraining program aimed at reducing organizational inefficiencies. Both OCD and control participants who underwent training improved more in organization and memory than participants who did not receive organizational training. The authors concluded that in OCD organizational capacities can be augmented. They however did not study the effects of this training on OCD symptoms. Second, in a promising RCT on the effects of a cognitive training program to improve organizational skills in 15 OCD patients compared to 15 controls, Park et al. [59] found that visual memory functions and obsessive-compulsive symptoms improved more in the treated group than in the control group.

## Methods/design

The study design is reported in line with the SPIRIT 2013 Statement (Standard protocol Items: Recommendations for Interventional Trials) [60]. The Medical Ethics Committee of the University Medical Centre Utrecht has approved the study (METc no. NL43751.041.13 v.03).

### Study aims

The primary goal of the current ongoing study is to examine the treatment enhancing effect of CRT on treatment as usual (TAU, guideline directed therapy) in AN and OCD. The secondary aim is to investigate the cost-effectiveness and budget impact of CRT. The third aim is to study which patient characteristics determine positive benefits from CRT-based treatment enhancement. More specifically, we study several moderators and mediators of response (Table 1).

**Table 1:** Measures to assess screening, outcome, mediator and moderator variables

Function	Measure	Instrument	T0	T1	T2	T3
Screening	Diagnostic status	SCID	X			
Primary outcome	AN severity	EDE-Q	X	X	X	X
	OCD severity	Y-BOCS	X	X	X	X
Secondary outcome	OCD severity	OCI-R	X	X	X	X
	Quality of life	EQ-5D	X	X	X	X
		ED-Qol ^c^	X	X	X	X
		OCD-Qol ^b^	X	X	X	X
	Daily functioning	WSAS	X	X	X	X
	Healthcare utilization and production loss	TIC-P	X	X	X	X
	Depression	BDI-II	X	X	X	X
	General anxiety	BAI	X	X	X	X
Mediators	Cognitive flexibility	Stroop	X	X	X	X
		D-Flex	X	X	X	X
		TMT	X	X	X	X
	Response inhibition	SSRT	X	X	X	X
	Central coherence	GEFT	X	X	X	X
	Intolerance of uncertainty	IUS	X	X	X	X
	Perfectionism	CPQ	X	X	X	X
	Self-efficacy	PEPPI-5	X	X	X	X
	Behaviour inhibition/activation	BISBAS	X	X	X	X
	Therapeutic engagement	SRS ^a^	X			
Moderators	Demographics, e.g. sex, age, education level	Interview	X			
	Diagnostic status	SCID-I	X			
	Obsessive compulsive personality disorder	SCID-II-OCPD	X			
	Autism	AQ	X			
	ADHD	CAARS-S:SV ^b^	X			
	Cognitive flexibility	ID/EDS ^b^	X			
	Planning ability	BADS-Zoo-map	X			

*EDQ* Eating Disorder Examination Questionnaire, *Y-BOCS* Yale-Brown Obsessive Compulsive Scale, *OCI-R* Obsessive-Compulsive Inventory-revised, *EQ-5D* EuroQuol, *ED-Qol* Eating Disorder Quality of life questionnaire, *OCD-Qol* Obsessive-Compulsive Disorder Quality of life questionnaire, *WSAS* Work and Social Adjustment Scale, *TIC-P* Trimbos/iMTA Questionnaire for Cost Associated with Psychiatric Illness, *BDI-II* Beck Depression Inventory, second revision, *BAI Beck* Anxiety Inventory, Stroop Stroop task, *D-FLEX* Detail and Flexibility Questionnaire, *TMT* Trail making test, *SSRT* Stop Signal Reaction Task, *GEFT* Group embedded figures Test, *IUS* Intolerance of uncertainty scale, *CPQ* Clinical perfectionism questionnaire, *PEPPI-5* Perceived Efficacy in Patient-Physician Interactions, *BISBAS* Behavioural Inhibition and Behavioural Activation Scales, *SRS* Session Rating Scale, *SCID-I* Structured Clinical Interview for DSM-IV-TR Axis I disorders, *SCID-II* Structured Clinical Interview for DSM-IV-TR Axis II disorders, section obsessive-compulsive personality disorder, *AQ* Autism-spectrum Quotient, *CAARS-S:SV* Connors’ Adult ADHD Rating Scales, self report, short version, *ID/EDS* Intra dimensional-/extra dimensional shift paradigm, *BADS-Zoo* map Behavioural Assessment of the Dysexecutive Syndrome, Zoo Map

^a^ after each CRT/SAT session

^b^ OCD Only

^c^ AN Only

Our research questions read as follows:

1. To what extend does CRT prior to TAU for AN and CBT for OCD enhance treatment outcome?
2. To what extend is addition of CRT to TAU more cost-effective?
3. What are moderators of treatment-enhancing effect of CRT in AN and OCD?
4. What are mediators of treatment-enhancing effect of CRT in AN and OCD?

### Design

**Fig. 1:**
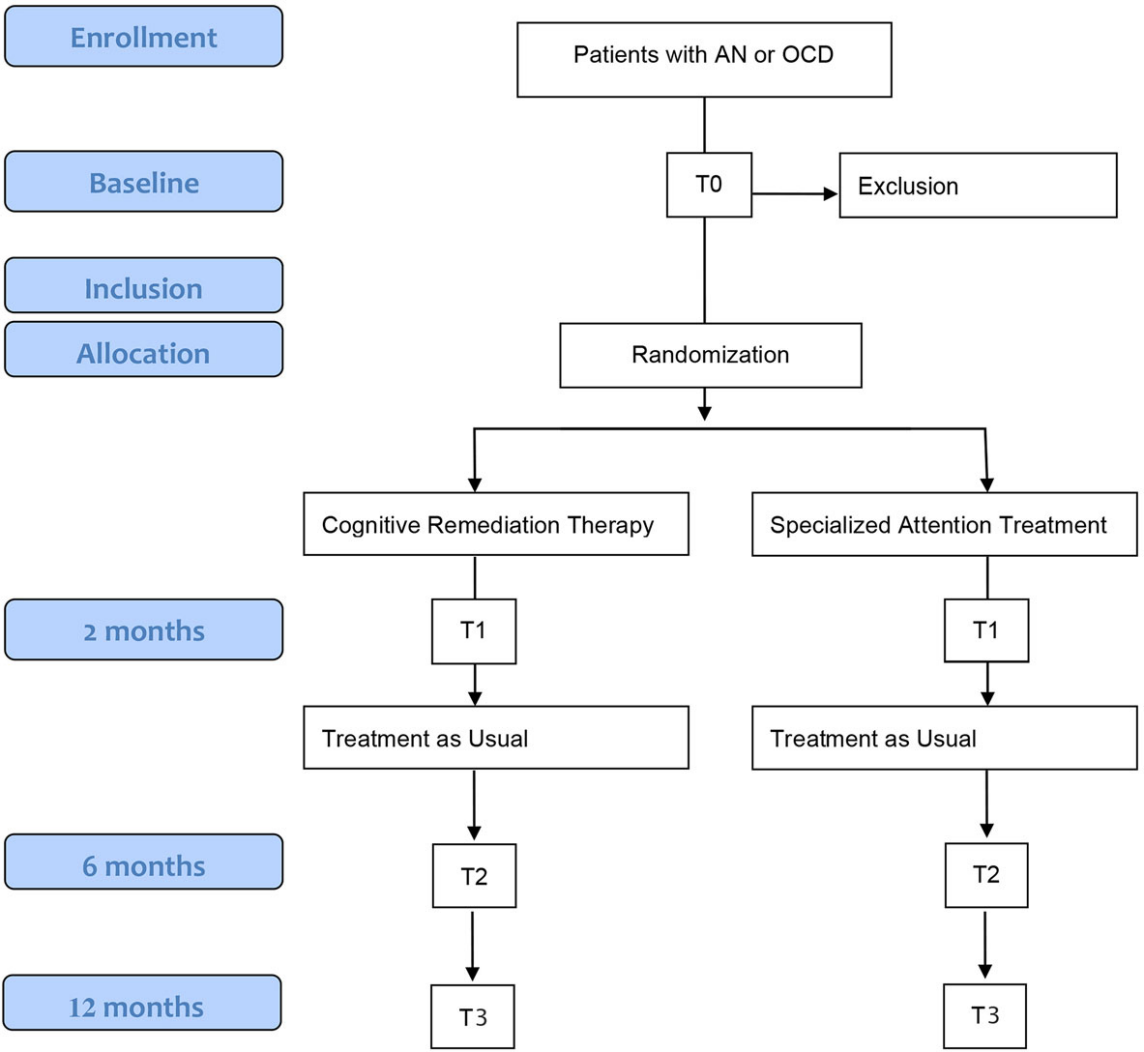
Study design

Figure 1 provides an overview of the study design. In a randomized controlled multicentre trial, we compare two treatment arms, i.e. CRT and, in the control condition, Specialized Attention Therapy (SAT), both delivered in 10 twice weekly sessions as an adjunct to TAU for AN and OCD. Patients are assessed at baseline (T0), directly following CRT or SAT completion and prior to symptom based TAU (T1), at 6 months after T0; (T2), and at 12 months after T0 (T3). All patients receive both oral and written information and are enrolled after they have provided written informed consent.

### Participants

Patients aged between 18 and 60 years are recruited from four Dutch tertiary clinics specialized in the treatment of anxiety and eating disorders. Patients diagnosed with AN (both restrictive and binge/purge subtype) are recruited from Altrecht Eating Disorders Rintveld, Zeist, and Rivierduinen Eating Disorders Ursula, Leiden, and patients with OCD from Altrecht Academic Anxiety Centre, Utrecht, and Overwaal Centre for Anxiety Disorders, Nijmegen. Most of the patients referred to these centres suffer from moderate to severe AN or OCD.

Eligible are patients whose AN (or eating disorder not otherwise specified, clinically referred to as AN) or OCD diagnosis is consistent with the Diagnostic and Statistical Manual of Mental Disorders, fourth edition text revision (DSM-IV-TR ).

The criteria for exclusion are: severe neurological illness (including a history of seizures, stroke, or Parkinson’s disease), severe comorbid psychiatric disorder (clinical significant major depressive disorder, current acute phase of bipolar disorder, current psychosis, substance dependence/abuse), intellectual impairment, defined as an IQ < 80, estimated with the Dutch Adult Reading Test (DART) [61], which is a Dutch version of the National Adult Reading Test (NART) [62], and inability to adequately speak or read Dutch. Current use of antidepressants and antipsychotics is allowed provided that dosages are kept constant during the experimental part of the study. Since benzodiazepines can dampen the effect of cognitive treatments [63], their use is restricted to a daily maximum dose (up to 20 mg for temazepam or an equivalent dose) taken as a sleep medication. On request, patients can withdraw their participation at any time.

Inclusion and exclusion criteria are verified with the aid of the structured clinical interview for DSM-IV-TR axis I disorders (SCID-I [64]), while for OCD patients to be enrolled, a Yale-Brown Obsessive-Compulsive Severity Scale score ≥16 is required [65, 66]. In all patients comorbidity with either OCD or AN is allowed.

### Procedure and randomization

All participating centres adhere to standardized intake and screening procedures. The therapist performing the screening informs patients meeting the inclusion criteria about the study and ask their permission to have a research assistant contact them by phone or in person to invite them to participate in the trial, after which interested patients are sent or given a brochure describing the study. At least 1 week later baseline assessments are carried out after written informed consent is obtained. After baseline assessments patients not meeting inclusion-criteria are excluded. Patients satisfying all the study criteria are subsequently randomized to one of the two study conditions. An independent research coordinator who is not involved in the therapies or assessments uses SPSS/IBM [67] to create a randomization sequence stratified by treatment centre with a 1:1 allocation using random block sizes of 4. Given the nature of the study design, all patients and therapists are aware of the treatment condition (CRT or SAT). Patients are informed that both treatment arms are being investigated as to their potential to enhance subsequent TAU. The therapists delivering CRT or SAT are instructed not to reveal the treatment condition to the research assistant conducting the assessments; unintended violation of assessor blindness is recorded.

### Measures

Table 1 provides a list of screening, outcome, mediator and moderator measures. Assessments to monitor treatment progress are performed at baseline (T0), after 2 months (post-CRT or SAT and prior to TAU) (T1), and 6 months after the baseline TAU (T2), and after 12 months (T3) This measurement will encompass the same instruments as used at T2. All patients are extensively re-evaluated using (validated or recommended) indices of treatment effect and costs, quality of life, and cognitive and behavioural flexibility, which will entail self-reports, structured clinical interviews, and paper-and-pen as well as computerized neuropsychological tests. All screening and outcome data are collected in a central database using SPSS/IBM [67].

### Screening measures

The Structured Clinical Interview for DSM-IV-TR Axis I disorders (SCID-I) [64] and the Structured Clinical Interview for DSM-IV-TR Axis II disorders (SCID-II) [68] are used to assess diagnostic status. The SCID-I [64] and SCID-II [68] are semi-structured interviews. Both interviews are organized by the DSM-IV classifications. The inter-rater reliability and validity of SCID-I and SCID-II interviews are fair to excellent depending the diagnosis [69] For the present study we used the full SCID-I and only the section on obsessive-compulsive personality disorder from the SCID-II.

### Outcome measures

The primary outcome measures are: 1) disease-specific psychopathology: Eating Disorder Examination Questionnaire (EDE-Q) [70] for AN severity; Yale-Brown Obsessive Compulsive Scale (Y-BOCS) [65, 66] for OCD severity.

The Eating Disorder Examination Questionnaire (EDE-Q) [70, 71] is the self-report version of the Eating Disorder Examination (EDE) [72], a semi-structured interview to evaluate ED psychopathology. The EDE-Q assesses attitudinal and behavioural aspects of EDs over a 28-day period. It has four subscales assessing concerns about shape, weight and eating, and restraint. The EDE-Q consists of subscale scores and a total scale score. The EDE-Q has excellent internal consistency (Cronbach α 0.78-0.93). The subscales have also excellent test-retest reliability over a 2-week period (Pearson r ranging from 0.81 to 0.94) [73].

The 10-item Yale-Brown Obsessive-Compulsive Severity Scale (Y-BOCS) [74] is a clinician-rated, semi-structured interview-based scale that is broadly used to assess obsessive-compulsive symptom severity. The scale has two parts, each with five questions, with each subscale assessing five aspects of OCD pathology: 1) time consumed, 2) degree of interference, 3) distress, 4) resistance, and 5) perceived control. The first subscale gives an obsession score (maximum: 20), the second a compulsion score (maximum: 20), and together yield a total score (maximum: 40). It has a strong internal consistency (Cronbach α .88-.91), inter-rater reliability (r 0.82–0.98), and test-retest reliability in clinical and nonclinical samples were excellent.

The secondary outcome measures are: 1) disease-specific psychopathology: Obsessive-Compulsive Inventory-Revised (OCI-R) [75], 2) quality of life and daily functioning: EuroQol (EQ-5D) [76], Eating Disorder Quality of life questionnaire (ED-QoL) [77], Obsessive Compulsive Disorder Quality of life questionnaire (OCD-QoL) [78], and Work and Social Adjustment Scale (WSAS) [79], 3) cost-effectiveness (direct and indirect health-related costs as evaluated by means of patients’ self-reported QoL ratings and medical and societal burden: Trimbos/iMTA Questionnaire for Costs associated with Psychiatric illness (TiC-P) [80], 4) general anxiety: Beck Anxiety Inventory (BAI) [81], and 5) Depression: Beck Depression Inventory, second revision (BDI-II) [82].

The Obsessive-Compulsive Inventory-revised (OCI-R) [75] evaluates the frequency, and distress experienced of OCD symptoms using six subscales: washing, obsessing, hoarding, ordering, checking, and neutralizing. The OCI-R has good psychometric properties [75, 83, 84] and is sensitive to the effects of treatment [85]. Internal consistency was adequate (obsessing α = .82, washing α = .86, checking α = .88, neutralizing α = .86, ordering α = .90, hoarding α = .90, total scale α = .81 ) and test-retest reliability was excellent (Spearman’s correlations between .79 and .91) [75].

The EuroQol (EQ-5D) [76] is a standardized self-report instrument gauging five dimensions: mobility, self-care, usual activities, pain/discomfort, and anxiety/depression. Each dimension is rated on three levels (no problems, some problems, and extreme problems). Further, the EQ-5D uses a visual analog scale from 0 to 100, on which the respondent marks his general well-being. The EQ-5D is used in a variety of studies to determine health status in a wide variety of patients.

The Eating disorder Quality-of-Life questionnaire (ED-QoL) [77] generates a disorder-specific self-rated quality-of-life score. It consists of 25 items assessing the influence of eating behaviours/body weight in four subscales: psychological, physical/cognitive, financial, and work/school. A total score is calculated as the average of the items of the four subscales. Higher scores indicate a lower quality of life. The ED-QoL has a good internal consistency (Cronbach α = 0.84–0.95), and test-retest reliability (intraclass correlations were .97 for the psychological subscale, .87 for the physical/cognitive subscale, .90 for the financial subscale, .14 for the work/school subscale, and .93 for the total score) [77].

The Obsessive-Compulsive Disorder Quality of Life (OCD-QoL) is a self-report questionnaire [78] gauging the influence of OCD on the respondent’s quality of life. It was adapted from the ED-QOL and thus also has four subscales: psychological, physical/cognitive, financial and work/school, whose 25 items are scored and interpreted similarly.

The Work and Social Adjustment Scale (WSAS) [79] examines to which extent the disorder has caused the patient functional impairment. A total score of 10 or below is normal, with scores of 10–20 indicating significant functional impairment but less severe clinical symptomatology, and scores of 20 and above suggest moderate to severe impairment or more severe psychopathology. The test-retest correlation for the total WSAS score was 0.73 and the scale has an acceptable to good internal consistency (Cronbach alpha 0.79–0.88).

The Trimbos/iMTA questionnaire for costs associated with psychiatric illness (TIC-P) [80] evaluates the utilization of medical services such as the number of contacts with the general practitioner and other care providers (e.g., medical specialists and paramedics) as well as medication used during the last 3 months. The costs associated with the experimental and control interventions delivered during this study will be calculated as recommended in the Dutch handbook on cost calculations in health care [86]. Reference unit prices of corresponding health-care services will be used and cost-utility calculated by relating the difference in direct medical costs per patient receiving CRT + TAU and SAT + TAU to the difference in terms of quality-adjusted life years (QALY) gained (cost-utility), yielding a QALY estimate.

The Beck Depression Inventory, second revision (BDI-II) [82] is a gold-standard self-report measure to assess the severity of depressive symptoms. The BDI-II comprises 21 categories of statements. Respondents are asked to choose the statement that describes the way they felt in the past 2 weeks. Each item is rated on a 4-point Likert-type scale ranging from 0 to 3, with higher scores indicating higher levels of depression. The maximum total score for all 21 items is 63. Score categories range from 0 to 13 (minimal depression), 14 to 19 (mild depression), 20 to 28 (moderate depression), and 29 to 63 (severe depression). The BDI-II has good psychometric properties [87]. The BDI-II shows good validity compared to the Hamilton Depression Rating Scale (Pearson’s r of 0.71). The inventory has high internal consistency (Cronbach α = .91) [88].

The Beck Anxiety inventory (BAI) [81] gauges symptoms of anxiety (such as trembling hands, dizziness, sweating) by means of 21 questions. Respondents indicate if and to what extent they have experienced any symptoms during the past week on a 4-point Likert scale ranging from 0 (not at all) to 3 (severely). The BAI discriminates well between anxious and non-anxious groups and is useful as screening measure for anxiety in a variety of clinical populations. The BAI has a high validity and high internal consistency (α .92).

### Mediators

The following variables will be tested as potential mediators of intervention response: 1) cognitive flexibility, using a Stroop task [89], including Delis-Kaplan Executive Function System (D-KEFS) card nr. 4, as well as Detail and Flexibility Questionnaire (D-Flex) [90], Trail Making Test (TMT) [91], 2) response inhibition: Stop Signal Reaction Task (SSRT) [92], 3) Central coherence: Group embedded figures test (GEFT) [93], 4) Intolerance of uncertainty: Intolerance of Uncertainty Scale (IUS) [94], 5) Perfectionism: Clinical Perfectionism Questionnaire (CPQ) [95], 6) Self-efficacy: Perceived Efficacy in Patient-Physician Interactions (PEPPI-5) [96], 7) Behavioural Inhibition and Behavioural Activation Scales (BISBAS) [97], and 8) therapeutic engagement Session Rating Scale (SRS) [98].

The Stroop-task was originally developed by Stroop [89] to easily measure selective attention and cognitive flexibility. The Stroop Color-Word Test has three components. Patients are first asked to name a series of colored words to reflect basic reading rate. They are next asked to name the color (red, blue, or green) of a bar. The final component comprises a Color-Word task in which the participant is shown the names of colors printed in conflicting ink colors (e.g., the word “green” in blue ink) and is asked to name the color of the ink rather than the word. For all tasks, patients are timed by the tester to determine the number of seconds needed to identify all the stimuli, and, for the interference condition, the number of uncorrected errors is recorded. The ‘Stroop Effect’ is calculated by subtracting the difference between the interference and color patch task times, where lower scores indicate better executive functioning. Average test-retest reliability for the Stroop task is high (*r* = 0.84) [99]. In the present study we use the Color-Word interference test from the D-KEFS test-battery [100].

The D-Flex [90] is a 24-item self-report scale that measures cognitive rigidity (difficulty with set-shifting/flexibility) and attention to detail (weak central coherence). The two subscales showed high internal consistency (Cronbach α 0.90–0.95), construct validity (as compared to relevant subscales of the AQ) was strong for cognitive rigidity (*r* = 0.72) but moderate for attention to detail (*r* = 0.26).

The Trail Making Test (TMT) [91] is used to evaluate set-shifting abilities. Originally a pen-and-paper test, more recently a computerized version has become available. Patients numerically or alphabetically connect circles on a page in a ‘ dot-to-dot ’ fashion (trail A), and then alternatively link numbers and letters, i.e. 1–A–2–B–3–C (trail B). Time taken to complete trail B (switching task) is the outcome measure of set-shifting ability.

When performing the computerized Stop Signal Task (SSRT) [92] patients need to rapidly select corresponding motor responses to left- or right-facing arrows appearing on a computer screen and attempt to inhibit responses when an auditory “stop signal” sounds. Using a tracking algorithm, the task estimates the time taken to internally suppress prepotent motor responses (stop-signal reaction times).

The Group Embedded Figures Test (GEFT) [101] is a paper-and-pencil task commonly used as a measure of central coherence. The time taken to find 18 simple shapes embedded in complex designs is recorded, with the main outcome being the total time needed to locate all hidden shapes, where shorter times are assumed to result from a strong local processing capacity or a bias toward detail.

The Intolerance of Uncertainty Scale (IUS) [94, 102, 103] was developed to assess reactions to ambiguous situations, uncertainty, and future events. The IUS has been investigated and validated in various populations to measure intolerance of uncertainty. The IUS includes 27 items relating to the idea that uncertainty is unacceptable, reflects badly on a person, and leads to frustration, stress, and the inability to take action. Respondents rate items on a 5-point Likert scale ranging from 1 = “not at all characteristic of me” to 5 = “entirely characteristic of me”. The Dutch version has high internal consistency for anxiety-disordered patients (Cronbach α = 0.94) and a satisfactory test-retest reliability (*r* = 0.79).

The Clinical Perfectionism Questionnaire (CPQ) assesses degrees of perfectionism. Respondents are presented with 12 items describing different expressions of perfectionism and asked to rate these on a 4-point Likert scale in relation to the last 28 days. Total score is the sum of all scores and range from 12 to 48, with higher scores indicating higher clinical perfectionism. There is evidence showing the CPQ to be a promising and reliable measure of clinical perfectionism. It has an acceptable internal consistency (Cronbach α = 0, 71) and test-retest reliability (*r* = 0.49–0.67) [95, 104].

With the Perceived Efficacy in Patient-Physician Interactions (PEPPI-5) [96] patients rate their sense of confidence or self-efficacy in regard to their interactions with their physician on a 5-point Likert scale ranging from ‘not at all confident’ (1) to ‘very confident’ (5). The instrument has been translated and validated for use in the Netherlands [105]. The range of possible scores is 5 to 25, with 25 reflecting highest-level self-efficacy. The PEPPI-5 has a high internal consistency (Cronbach alpha 0.92) and a fair test-retest reliability (intra-class correlation coefficient 0.68).

The Behavioural Inhibition and Behavioural Activation Scale (BISBAS) [97] is a 20-item instrument designed to measure behavioural inhibition (i.e., concern over and reactivity to aversive events) and behavioural activation (i.e., responsiveness to incentives, drive, and fun seeking). Patients respond to the items using a 4-point scale, ranging from 1 (quite untrue) to 4 (quite true). After reverse scoring of two items on the BIS, subscale scores are computed by summing the scores corresponding to items on the BIS scale (7 items, range 7–28, α .74). The BAS-scale (13 items, range scores 13–52) consists of the reward responsiveness scale (5 items, α .73), the drive scale (4 items, α .76), and the Fun Seeking scale (4 items, α .66). Test-retest correlations over an 8 week period are satisfactory (r from 0.59 to 0.69). The BIS correlates moderately highly with measures of trait anxiety, negative affect, and negative temperament. The BAS correlates positively with extraversion, positive affect, and positive temperament.

The Session Rating Scale (SRS) [98] is a short, 4-item, self-report questionnaire probing therapeutic alliance. After each session patients are asked to give their impressions of the preceding session using a VAS. They rate their bond with the therapist, agreement on therapy goals and therapy tasks, and their confidence in the collaboration, where ratings can range from “there was something missing in the session today” to “Overall, today’s session was right for me”. The Dutch translation of the SRS has been shown to have a strong internal consistency (Cronbach alpha 0.85–0.95) and adequate test-retest reliability [106], although in the Dutch study correlations with outcome and concurrent validity were lower than expected.

### Moderators

The following variables will be tested as potential moderators of intervention response: 1) demographic variables (e.g. sex, age, educational level), 2) general psychopathology: Structured Clinical Interview for DSM-IV-TR Axis I disorders (SCID-I) [64], Structured Clinical Interview for DSM-IV-TR Axis II disorders, section obsessive-compulsive personality disorders (SCID-II-OCPD) [68], 3) Autism: Autism-spectrum Quotient (AQ) [107], 4) Attention-deficit hyperactivity disorder: Connors’ adult ADHD Rating Scales CAARS-S:SV) [108], 5) Cognitive flexibility: Intra dimensional-/extra dimensional shift paradigm (ID/EDS) [109], and 6) Planning ability: Behavioural Assessment of the Dysexecutive Syndrome, Zoo MAP (BADS-Zoo map) [110]. The SSRT, IUS, and BADS-Zoo map will be taken for OCD only.

The autism-spectrum quotient (AQ) [107] is a self-report questionnaire that assesses traits associated with the autistic spectrum [111]. It consists of five 10-item subscales (social skills, attention switching, communication, imagination, attention to detail). Example items of the AQ are: “I find myself drawn more strongly to people than to things” and “Trying to imagine something, I find it easy to create a picture in my mind”. Respondent rate each item on a 4-point Likert scale from “definitely agree” to “definitely disagree”. A score of ≥ 32 is indicative of an autism spectrum disorder. The index’s test-retest reliability is good (*r* = .60 to .81) and internal consistency is acceptable (α = .71) [112].

The Connors’ Adult ADHD Rating Scales self-report, short version (CAARS-S:SV) [108] comprises 30 items on the presence of ADHD symptoms to be rated on a 4-point Likert scale ranging from 0 (not at all), to 3 (very much). The maximum score for al 30 items is 90. The CAARS-S:SV consists of 5 subscales (inattention/memory problems, hyperactivity/restlessness, impulsivity/emotional lability, problems with self-concept, and ADHD-index). The internal consistency is good, ranging from α = .80 to .89 and it has an acceptable test-retest reliability (*r* = 0.58 to 0.77).

The Intra-dimensional/extra-dimensional shift task (ID/EDS) [109] is part of the Cambridge Neuropsychological Test Automated Battery (CANTAB). The version used in the current trial is a replication of the original task. The ID/EDS is a reliable and valid computerized task, which, like the TMT, probes set-shifting ability [113–115]. The paradigm involves nine-stages of a visual discrimination task with multidimensional stimuli. Two stimuli are displayed, and feedback is provided which stimulus is correct. To pass to the next stage, six consecutive correct responses are required within 50 trials; if not, the task ends. The rule for correct responding is modified at the start of each task stage.

The Zoo Map is part of the Behavioural Assessment of the Dysexecutive Syndrome test battery (BADS) [110] and consists of two parts. In the first part, patients are instructed to plan their route through a map of a zoo, visiting a selection of locations while actively disregarding others. While planning the route, patients also instructed to obey certain rules. The second part consists of the same map with the same locations that have to be visited, but this time, instructions are provided about the precise order in which the locations must be visited. Therefore, in contrast to the first part, the second part consists of a highly structured setting that strongly reduces the involvement of planning abilities. The BADS-Zoo map subtest has been proven to be a valid indicator of planning ability in a heterogeneous patient sample [116].

### Power and sample-size calculation

In accordance with Cohen [117], we calculated the sample sizes needed to detect treatment-specific differences: CRT + TAU vs. SAT + TAU, for which we used software by G-power [118].

We based effect-size calculations on Dingemans’ first RCT [57] comparing CRT + TAU with TAU alone for eating disorders in which they obtained large effect sizes for reduction in eating disorder pathology (Cohen’s d: 0.99). Given the control condition in this study we take into account a smaller effect-size. In the case of two-tailed MANOVAs, repeated measures and four within-between interactions (2 groups: AN and OCD), two conditions (CRT + TAU vs. SAT + TAU), three measurements (baseline, and 2, and 6 months after baseline), with alpha = 0.05, a sample size of 113 patients is needed to have a power of at least 0.80 to detect an effect size f(V) of 0.25. Although the dropout rate in the previous CRT studies of Dingemans was low (*n* = 7 out of 82 patients), we have opted to include an additional 15 patients to compensate for attrition, totalling 128 patients in total (64 CRT + TAU and 64 SAT + TAU).

### Interventions

CRT and SAT are both delivered in 10 bi-weekly sessions directly preceding TAU. As AN patients will need to be closely monitored and given nutritional advice throughout the study, the relevant treatment components will be part of the first treatment phase in this group. Other components of (psychological) treatment will not be offered until CRT/SAT has been completed.

### Cognitive remediation therapy

In this trial, CRT is based on the Dutch translation of the original manual by Kate Tchanturia [119] for both patient groups. Comprising 10 twice weekly 45-min sessions, CRT is delivered by psychologists, clinical nurses, and psychology students (at the MSc level), all having been trained by experienced CRT therapists (UD, BvP, LS, DC) who themselves were trained by Dr Kate Tchanturia, expert on CRT for eating and weight disorders. Using a range of cognitive lab-based exercises, CRT aims at improving patients’ set-shifting, cognitive flexibility, global information processing skills, and at reducing perfectionism. Table 2 lists the within-session exercises used in the RCT.

**Table 2:** CRT exercises

Central coherence tasks	Maps Summarize a letter Hidden words Word-search task Embedded word task
Set shifting tasks	Card Stack Visual illusions Stroop material Switching attention task Switching Time Zones Alphabet task
Perfectionism tasks	Estimation task
Strategy tasks	How to plant a sunflower Complex figure task

For a more detailed description of the exercises, see Tchanturia et al. [119]. With respect to carrying out the exercises, awareness of dysfunctional thinking styles is enhanced. Patients are encouraged to find out how these thinking styles affect their daily lives, and from session 2 onward, homework assignments are given comprising behavioural exercises that are closely linked to real-life skills and tasks to thus stimulate patients to practice more flexible behaviours in their everyday lives.

### Specialized Attention Therapy (SAT)

SAT has been designed as the control intervention specifically for this trial. SAT is delivered by the same health professionals delivering CRT. The SAT session structure (10 twice weekly, 45-min sessions and homework assignment) is the same as in CRT. However, during SAT, cognitive flexibility, central coherence, global information processing skills, awareness of thinking styles, and reducing perfectionism are not addressed. Instead, after each task patients are asked their opinion on whether they (dis)liked the task. As a treatment rationale, patients are explained that SAT aims at helping them to focus on positive experiences by means of tasks and exercises, where each session–again in line with CRT–comprises different exercises. For an overview of the exercises practiced in SAT, see Table 3; for a more detailed description of the exercises, the SAT treatment manual is available upon request [120].

**Table 3:** SAT exercises

Motor tasks	Djenga Mikado Leaning Tower of Pisa Operation Game
Luck tasks	Game of the Goose Ludo Snakes and ladders
Collaboration task	Drawing a picture together
Picture tasks	Spot the differences Viewing a picture
Verbal tasks	Last letter-first letter Incomplete sentences task Poems
Additional tasks	Listening to music Analogies Categories

From session 2 onward, patients are encouraged to engage in similar activities at home that they enjoy.

### Treatment as usual

After completion of either CRT or SAT, patients receive TAU. The content of TAU comprises all the essential elements recommended in the Dutch guidelines on eating disorders and OCD, which are closely related to the international guidelines [121, 122].

TAU for patients with AN consists of normalization of eating behaviours (weight gain), discussion of daily problems, goals, psycho-education, and for a subgroup psychomotor therapy, art therapy, social skills training, family-therapy or cognitive behavioural therapy. Psychiatric- and/or medical consultation and pharmacotherapy is given where needed.

TAU for OCD patients comprises CBT delivered in 20 to 30 weekly or twice weekly 45–90 min sessions in which exposure with response prevention forms a key element, consistent with the multidisciplinary guidelines for anxiety disorders (OCD chapter) [121]. In line with the literature [123, 124], the OCD protocol is flexible, i.e., patient-tailored with regard to the time spent on each component, accounting for differences in session and treatment duration. The therapy encompasses a) psychoeducation, b) cognitive therapy, c) exposure in vivo with response prevention, d) psychiatric consultation, and, in case of severe symptoms, pharmacotherapy with SSRIs, venlafaxine, clomipramine and/or atypical antipsychotics. The protocol allows for one or two sessions in which the principles of CBT are explained and one or two sessions in which an inventory is made of all anxiety/tension-provoking situations that give rise to compulsions and a construction of a list of compulsion triggers. All patients receive in- and between session exposure to anxiety-provoking thoughts and situations, coupled with prolonged response prevention tailored to their individual symptoms.

### Treatment fidelity

To ensure treatment fidelity for CRT and SAT, the best practices and recommendations from the NIH Behaviour Change Consortium [125] are followed. The number, duration, and frequency of sessions, as well as patient-therapist contact times are kept the same for CRT and SAT. All patient-therapist contacts are formally scheduled to ensure fixed durations. All treatment sessions are audio- or videotaped and an external checker not involved in the trial rates treatment fidelity in a random selection of 10% of the recorded sessions. To evaluate, guide, and approve interventions, and to constrain ‘therapist drift’, all therapists are supervised by expert professionals, and have at least monthly intervision-sessions. After every session, the therapist completes a brief questionnaire to record treatment stage and components delivered. Deviations from the protocol are reported to the supervisor.

### Analyses

Baseline differences between the CRT and SAT (control) groups and between the participating centres are investigated using χ^2^ tests for categorical variables and ANOVAs for continuous variables. Additional exploratory analyses are conducted separately for AN and OCD.

The data for the primary AN and OCD outcome indices are analysed using linear mixed models (LMM) with the baseline value as a covariate. The mixed models analysing the general efficacy of CRT include a random term for the intercept and fixed terms for condition, time contrasts (T0–T1, T0–T2, T0–T3, T1–T2, T1–T3), and the interactions between condition and time contrasts.

In order to investigate moderators of short- and long-term treatment response, time contrasts are created (T0–T1, T0–T2, T0–T3, T1–T2, T1–T3) by means of dummy coding. Potential continuous moderator variables are standardized. Interactions between condition and the potential moderators are entered into the equation. All analyses on therapy effect modificators are of an exploratory nature. The moderator and mediator analyses are calculated with 5000 bootstraps [126].

To assess the cost-effectiveness of CRT + TAU versus SAT + TAU for ED and OCD, all relevant costs and effects are taken into account. Disease progression is described in terms of transitions between ‘states’, where a patient’s ‘state’ can shift in either direction or remain constant. Estimations of state-transition rates are based on the data from the ongoing trial and the literature, which modelling approach serves to help us assess future scenarios. Costs and effects are calculated in accordance with recommendations in the Dutch guideline for economic evaluation of health care. As a primary assessment we apply a costs-utility analysis that yields a cost per quality-adjusted life years (QALY) outcome, a common cost-effectiveness measure that allows comparison with economic evaluations of other diseases and interventions. The uncertainty estimations are computed using bootstrapping and the results expressed in an acceptability curve. If no intervention is effective, a cost-minimization calculation is performed.

## Discussion

Inefficiencies in cognitive flexibility and central coherence are problematic in psychiatric disorders. CRT aims at addressing these inefficiencies and thus enhancing treatment outcome. To our knowledge, this is the first randomized controlled trial using a control condition evaluating the efficacy and effectiveness of CRT as a treatment enhancer preceding TAU for AN, and the first study to investigate CRT in OCD, moreover taking cost-effectiveness of CRT in AN and OCD into account. This study enables us to establish whether the anticipated treatment-enhancing effect of CRT is achieved by improvement of the patients’ cognitive flexibility. With this transdiagnostic approach, we will additionally learn whether CRT challenges a diagnosis-specific problem or taps shared underlying mechanisms for AN and OCD.

Another strength of our trial is that CRT effectiveness is investigated in established clinics specialized in the treatment of patients with severe symptoms.

A potential limitation of this trial is the fact that we have not tested the validity of SAT as a control condition, i.e. a condition that lacks the potential working mechanisms of CRT . Further, the credibility of SAT as an alternative treatment to CRT has not been tested. After baseline assessment, patients are told which intervention they receive (CRT or SAT). Although they are not told that CRT is the therapy-enhancer being investigated and that SAT is offered as a control intervention, patients might be able to ‘guess’ which therapy entails the control condition, potentially biasing treatment expectancy and credibility.

## Abbreviations

ADHD: Attention deficit hyperactivity disorder
AN: Anorexia nervosa
AQ: Autism-spectrum quotient
BADS: Behavioural Assessment of the Dysexecutive Syndrome
BAI: Beck Anxiety Inventory
BDI-II: Beck Depression Inventory, second revision
BISBAS: Behavioural Inhibition and Behavioural Activation Scales
BvP: Boris van Passel
CAARS-S-SV: Connors’ Adult ADHD Rating Scales, Self-report, Short Version
CANTAB: Cambridge Neuropsychological Test Automated Battery
CBT: Cognitive Behaviour Therapy
CPQ: Clinical Perfectionism Questionnaire
CRT: Cognitive remediation therapy
DART: Dutch Adult Reading Test
DC: Daniëlle Cath
D-FLEX: Detail and Flexibility Questionnaire
D-KEFS: Delis-Kaplan Executive Function System
DSM-IV-TR: Diagnostic and Statistical Manual of Mental Disorders, fourth edition text revision
ED: Eating disorder
EDE-Q: Eating Disorder Examination Questionnaire
EDQoL: Eating Disorder Quality of Life questionnaire
EQ-5D: EuroQoL five dimensions questionnaire
GEFT: Group embedded figures test
IBM: International Business Machines
ID/EDS: Intra dimensional-/extra dimensional shift paradigm
iMTA: Institute for Medical Technology Assessment
IUS: Intolerance of Uncertainty Scale
LMM: Linear mixed models
LS: Lot Sternheim
METc: Medical Ethical Committee (in Dutch: Medisch Ethische Toetsingsingscommissie)
MSc: Master of Science
NART: National Adult Reading Test
NIH: National Institutes of Health
OCD: Obsessive-compulsive disorder
OCDQoL: Obsessive-Compulsive Disorder Quality of Life questionnaire
OCI-R: Obsessive-Compulsive Inventory-Revised
OCPD: Obsessive-compulsive personality disorder
PEPPI-5: Perceived Efficacy in Patient-Physician Interactions
QALY: Quality Adjusted Life Years
Qol: Quality of life
RCT: Randomized controlled trial
SAT: Specialized Attention Therapy
SCID-I: Structured clinical interview for DSM-IV-TR axis I disorders
SCID-II: Structured Clinical Interview for DSM-IV-TR axis II disorders
SPSS: Statistical Package for the Social Sciences
SRS: Session Rating Scale
SSRI: Selective serotonin reuptake inhibitor
SSRT: Stop Signal Reaction Task
TAU: Treatment as usual
TIC-p: Trimbos/iMTA questionnaire for Costs associated with Psychiatric illness
TMT: Trail Making Test
UD: Unna Danner
WSAS: Work and Social Adjustment Scale
Y-BOCS: Yale-Brown Obsessive Compulsive Scale
